# The C-reactive protein/albumin ratio as a nutritional biomarker in maintenance hemodialysis patients: a cross-sectional study of malnutrition-inflammation status assessment

**DOI:** 10.3389/fmed.2025.1637536

**Published:** 2025-11-24

**Authors:** Mingzhu Li, Zheng Jiang, Dongmei Zhang

**Affiliations:** 1 Department of Nephrology, The Affiliated Hospital of Southwest Medical University, Luzhou, China; 2 Sichuan Clinical Research Center for Nephropathy, Luzhou, China

**Keywords:** hemodialysis, malnutrition-inflammation score, protein-energy wasting, C-reactive protein to albumin ratio, end-stage renal disease

## Abstract

**Background:**

The C-reactive protein to albumin ratio (CAR) is a biomarker associated with various diseases; however, its significance in maintenance hemodialysis (MHD) patients remains unclear. This study sought to explore the relationship between the Malnutrition-Inflammation Score (MIS) and CAR in this population.

**Methods:**

In a cross-sectional study, 231 adult MHD patients were enrolled and categorized into high (*n* = 98) and low (*n* = 133) MIS groups based on an optimala cutoff value of 7. Detailed analyses were conducted on the MIS, biochemical parameters, and other biomarker ratios to assess their relationships.

**Results:**

Significant differences were observed in albumin, CRP, CAR, and RAR levels (all *p* < 0.05). A significant association was observed between CAR and MIS with MIS (OR 1.05, *p* < 0.001). The area under the receiver operating characteristic curve (AUROC) for CAR in identifying nutritional and inflammatory risk was 73.85%, with an optimal cutoff value of 2.158. A non-linear relationship was also identified between MIS and CAR.

**Conclusion:**

CAR is independently associated with the MIS in MHD patients and may serve as a valuable biomarker, underscoring its potential value for clinical nutritional management of this patient population.

## 1 Introduction

Hemodialysis (HD) is the primary renal replacement therapy for patients with end-stage renal disease (ESRD). Despite its effectiveness, patients on maintenance hemodialysis (MHD) often develop a series of complications in the later stages of treatment. Among these, malnutrition and inflammation are common and interrelated complications that significantly affect patient outcomes. These conditions not only independently elevate the risk of adverse cardiovascular events and all-cause mortality but also interact through complex pathophysiological pathways to form a vicious cycle, further deteriorating prognosis (1, 2).

It is now understood that the chronic inflammatory state in HD patients is primarily driven by factors such as the accumulation of uremic toxins, repeated exposure to biocompatibility-mismatched dialyzers, and heightened susceptibility to infections (3–5). Malnutrition, a prevalent condition in this population, arises through mechanisms including inadequate protein intake, amino acid loss during dialysis, intestinal absorption dysfunction, and increased metabolic demands (6–10). Notably, malnutrition itself compromises immune function, thereby elevating the risk of infection and further exacerbating the inflammatory response. For example, hypoalbuminemia reduces the transport of antioxidants (e.g., glutathione), promoting reactive oxygen species (ROS) accumulation that activates the nuclear factor-κB (NF-κB) pathway, thereby exacerbating inflammation (11, 12).

In recent years, protein-energy wasting (PEW), defined as the loss of body protein mass and energy reserves in HD patients, has emerged as a significant clinical concern characterized by low serum albumin, transferrin, or cholesterol levels, and unintentional weight loss (13). PEW represents a prevalent issue among individuals undergoing HD, with improved nutritional status linked to significantly reduced mortality and additional clinical benefits (14, 15). Consequently, nutritional management is crucial in the therapeutic care of HD patients. To effectively identify nutritional risk, researchers recommend the use of multiple assessment tools, such as the Subjective Global Assessment (SGA), Malnutrition-Inflammation Score (MIS), and Geriatric Nutritional Risk Index (GNRI). These instruments have been validated as reliable methods for predicting patient outcomes (16–18). Among these, the MIS, which incorporates biochemical indices, anthropometric measurements, and subjective clinical evaluations, has shown the most robust predictive power for PEW (19).

However, traditional nutritional indices such as serum albumin (ALB) have significant limitations: First, due to its long half-life, ALB cannot accurately reflect real-time changes in nutritional status; Besides, a decline in ALB levels during inflammation primarily stems from synthesis inhibition and distribution abnormalities rather than simple nutritional deficiency, such that hypoalbuminemia can be regarded as an inflammatory marker (20, 21). Moreover, C-reactive protein (CRP), a classic inflammatory marker, is sensitive to inflammatory activity but does not directly assess nutritional status. Studies have demonstrated a significant negative correlation between ALB and CRP in HD patients (*r* = -0.311, *p* < 0.01) (22), suggesting that these two markers collectively reflect a combined nutritional and inflammatory status.

While existing scores exhibit good performance in predicting PEW, their calculation remains complex. In recent years, composite biomarkers such as the C-reactive protein to albumin ratio (CAR), red blood cell distribution width to albumin ratio (RAR), and platelet to albumin ratio (PAR) have attracted significant interest. These novel biomarkers have demonstrated predictive potential in diverse diseases, including renal cell carcinoma (23), sepsis (24), coronary atherosclerotic heart disease (25), diabetes (25), and IgA nephropathy (26). Building upon the above findings, this study aims to systematically investigate the efficacy of CAR as a biomarker for PEW in HD patients and to explore the predictive role of composite indices in assessing MIS.

## 2 Materials and methods

### 2.1 Data sources and study population

**FIGURE 1:**
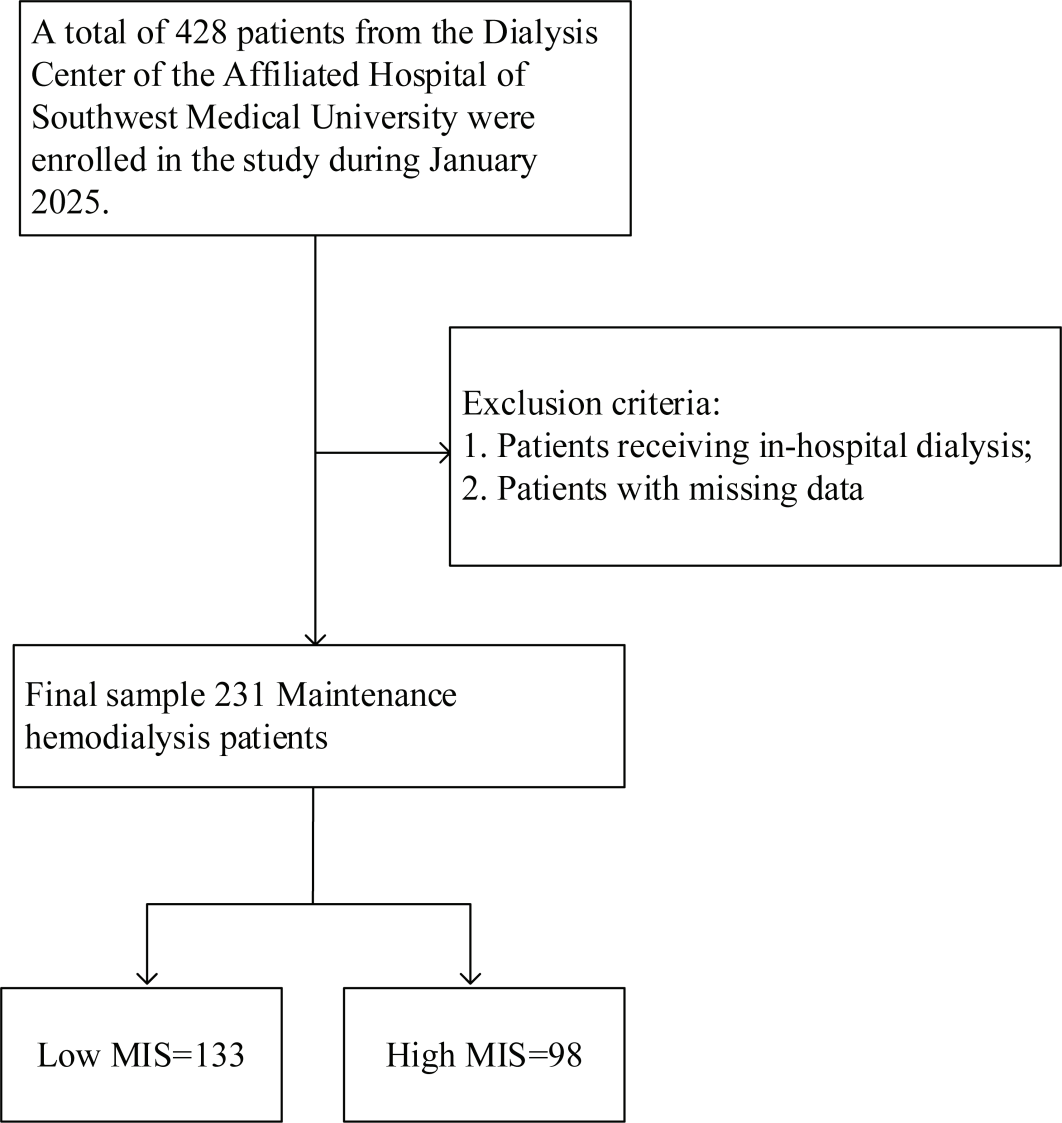
Research flowchart. A total of 428 patients undergoing maintenance hemodialysis (MHD) were initially assessed for eligibility. According to the predefined inclusion and exclusion criteria, 197 patients were excluded, resulting in 231 patients being included in the final analysis. These patients were categorized into a low MIS group (*n* = 133) and a high MIS group (*n* = 98) based on a cutoff score of 7. MIS, Malnutrition Inflammation Score.

This study enrolled patients who received maintenance hemodialysis at the Affiliated Hospital of Southwest Medical University in January 2025. The inclusion criteria were patients with a definitive diagnosis of stage 5 chronic kidney disease and those who required long-term maintenance hemodialysis treatment. Exclusion criteria encompassed severe infections, surgical procedures, receiving in-hospital dialysis, missing data, and pregnancy. Ultimately, a total of 231 eligible patients were enrolled in the study (Figure 1).

### 2.2 Malnutrition-inflammation score

The MIS is a comprehensive scoring system utilized for the assessment of malnutrition and inflammatory status. The MIS comprises ten components, encompassing weight change, dialysis duration, coexisting diseases, Body Mass Index (BMI), laboratory indices, and subjective signs. Each scoring item is scored on a scale of 0 to 3, with 0 (indicating a normal condition) to 3 (indicating a severe state of malnutrition and inflammation). During the scoring process, all subjective assessments of the MIS are conducted by two physicians. In cases of discrepancy in the scores, a second round of scoring was conducted following a discussion. Based on established literature, MIS ≥ 7 was defined as PEW (27, 28).

Two experienced physicians independently evaluated the subjective components of the MIS. Prior to the study, both physicians were trained using the standard MIS assessment guidelines to ensure a consistent understanding of the criteria, particularly for subjective items. All scoring was performed blinded to the patients’ laboratory results, including CRP and albumin levels. Any discrepancies in scores were resolved through discussion until a consensus was reached. Inter-rater reliability was assessed using Cohen’s kappa statistic, which showed very good agreement (κ = 0.85).

### 2.3 Measurement of CAR, RAR, and PAR

Inflammation markers and nutritional parameters were used to identify correlations for predicting the nutritional status of MHD patients. These composite indicators are calculated as follows: CAR = CRP level (mg/L)/albumin level (mg/dL), RAR = red blood cell distribution width (fL)/albumin level (mg/dL), PAR = platelet (*10^9^/L)/albumin level (mg/dL).

### 2.4 Statistical analysis

In this study, the enrolled patients were divided into a high-MIS group and a low-MIS group based on a cutoff value of 7, with the distribution of baseline data compared between the two groups. Descriptive statistics were reported as frequencies and proportions for categorical variables and as medians (IQR) or means (SD) for continuous variables. Categorical variables were presented as percentages (%). According to the normality test and analysis of variance, an independent samples *t*-test, Mann-Whitney U test or chi-square (χ^2^) test was used to compare the differences between participants with high MIS and low MIS. The univariate logistic regression model was used to evaluate the odds ratio (OR) of the association between variables and nutritional risk, as well as the 95% confidence interval (CI). Variables with a *p* < 0.05 were included in the multivariate analysis. Receiver operating characteristic (ROC) curve analysis was employed to explore the predictive performance of CAR, CRP, and ALB in predicting MIS values. The Youden index was utilized to determine the optimal cut-off point for CAR prediction, and the restricted cubic spline (RCS) plot was used to evaluate the association between CAR and MIS scores. All probabilities were calculated using two-tailed tests, with the significance level set at 0.05.

Statistical analysis was performed using SPSS (Version 24.0). The ROC curves were generated by Graph Prism 8.0. The RCS curves were plotted using R (4.4.3) and Zstats v1.0.^1^

### 2.5 Ethical approval

Ethical approval for this cross-sectional study was granted by The Affiliated Hospital of Southwest Medical University (KY2025167). The need for informed consent was waived due to the use of de-identified patient data.

## 3 Results

### 3.1 Patients

A total of 428 patients undergoing MHD were initially screened. Based on the established inclusion and exclusion criteria, 231 patients were ultimately selected for analysis (Figure 1). Patients were categorized into two groups according to their MIS: a low MIS group (*n* = 133, 57.6%) and a high MIS group (*n* = 98, 42.4%). The baseline demographic and clinical characteristics of these groups are detailed in Table 1. Both groups exhibited comparable characteristics in terms of dialysis vintage, dry weight, and BMI (*p* > 0.05). However, patients in the high MIS score group were significantly older than those in the low MIS score group (*p* < 0.05). Regarding laboratory indicators, the low MIS score group demonstrated significantly lower levels of white blood cells, monocytes, eosinophils, and CRP compared to the high MIS score group (*p* < 0.05). Conversely, the levels of transferrin, blood urea nitrogen, creatinine, uric acid, albumin, and prealbumin were significantly higher in the low MIS score group than in the high MIS score group (*p* < 0.05).

**TABLE 1:** Comparison of baseline characteristics between maintenance hemodialysis (MHD) patients with low and high malnutrition-inflammation score (MIS).

Variable	Low MIS (*n* = 133)	High MIS (*n* = 98)	t/Z/X^2^	*p*
Age	55(46–61)	60(53–71.5)	-4.084	< 0.001
Sex, n (%)				
Male	81	57	0.176	0.675
Famale	52	41		
Height (cm)	164(157–170)	158(161.5–168)	-1.104	0.269
Dialysis age (y)	5(2–8)	4(1.5–6.5)	-2.202	0.028
Dry weight (Kg)	60(52.7–69.0)	61.75(52.25–68.45)	-0.124	0.902
BMI	22.41(20.90–25.02)	22.78(20.55–26.14)	-0.219	0.827
Kt/V	1.38(1.21–1.63)	1.44(1.28–1.61)	-1.126	0.220
DM				
No	98	60	4.053	0.044
Yes	35	38		
RET–He (pg)	31.70(29.40–33.30)	30.95(28.90–32.90)	-1.338	0.181
Hemoglobin (g/dL)	11.05 ± 1.50	10.73 ± 1.38	1.668	0.097
White blood cell	5.80(4.87–7.14)	6.18(5.20–7.54)	-1.136	0.256
Lymphocyte (*10^9^/L)	1.04 ± 0.35	1.05 ± 0.37	-0.491	0.624
Monocyte (*10^9^/L)	0.46(0.35–0.58)	0.51(0.39–0.63)	-2.057	0.040
Neutrophil (*10^9^/L)	4.03(3.36–5.19)	4.40(3.50–5.38)	-1.092	0.275
RBC (*10^9^/L)	3.63(3.31–3.94)	3.56(3.22–3.92)	-0.803	0.422
Eosinophil (*10^9^/L)	0.21(0.13–0.31)	0.21(0.14–0.33)	-0.005	0.996
Platelet (*10^9^/L)	177(143–215)	179(134–219)	-0.354	0.724
MPV (fL)	10.30(9.70–10.90)	10.4(9.90–11.00)	-0.950	0.342
RDW (fL)	48.60(45.80–51.20)	50.55(46.35–54.00)	-2.922	0.003
MCH (pg)	30.40(29.05–31.70)	30.40(29.05–31.85)	-0.255	0.799
MCV (fL)	95.10(92.40–98.80)	96.80(92.45–100.30)	-0.901	0.367
Ferritin (ng/mL)	115.38(46.38–226.41)	144027(52.40–238.41)	-0.685	0.493
Transferrin (g/L)	1.70(1.09–2.19)	1.37(0.82–1.71)	-3.271	0.001
Serum iron (μmol/L)	11.10(8.60–14.25)	10.80(7.90–14.25)	-1.146	0.252
Urea nitrogen (mmol/L)	27.99(23.13–31.94)	24.56(19.96–28.76)	-3.501	< 0.001
Creatinine (μmol/L)	1073.29 ± 266.41	888.10 ± 254.57	5.211	< 0.001
Uric acid (μmol/L)	466.20(407.40–538.30)	421.80(363.80–483.65)	-3.002	0.003
Total protein (g/dL)	67.94 ± 4.63	66.31 ± 5.35	2.510	0.013
Albumin (g/dL)	3.96(3.83–4.12)	3.76(3.58–3.92)	-6.098	< 0.001
LDL–C (mmol/L)	2.10(1.59–2.76)	1.66(1.03–2.06)	-0.781	0.435
Total cholesterol (mmol/L)	3.53(2.88–4.30)	3.34(2.65–4.32)	-0.623	0.533
Triglyceride	1.70(1.17–2.44)	1.66(1.03–2.06)	-1.366	0.172
Prealbumin (g/L)	3.29 ± 0.66	2.84 ± 0.68	4.989	< 0.001
CRP (mg/L)	2.30(1.20–5.20)	8.25(2.35–15.1)	-6.157	< 0.001
CAR	0.56(0.31–1.31)	2.26(0.61–4.12)	-6.139	< 0.001
RAR	12.23(11.28–13.22)	13.62(12.46–15.06)	-5.598	< 0.001
PAR	44.62(36.72–54.24)	46.16(35.40–58.00)	-0.798	0.425

Data are presented as mean ± standard deviation, median (interquartile range), or number (percentage), as appropriate. Comparisons were made using the independent samples *t*-test, Mann-Whitney U test, or chi-square test. BMI, body mass index; DM, diabetes mellitus; RET-He, reticulocyte hemoglobin equivalent; RBC, red blood cell count; MPV, mean platelet volume; RDW, red cell distribution width; MCH, mean corpuscular hemoglobin; MCV, mean corpuscular volume; LDL-C, low-density lipoprotein cholesterol; CRP, C-reactive protein; CAR, C-reactive protein to albumin ratio; RAR, red cell distribution width to albumin ratio; PAR, platelet to albumin ratio. *p* < 0.05 was considered statistically significant.

### 3.2 Univariate and multivariable regression analyses

The results of the univariate and multivariate logistic regression analyses are presented in Table 2. The indicators with a *p* < 0.05 in the univariate logistic regression were included in the multivariate analysis. The variance inflation factor (VIF) was calculated to adjust for multicollinearity. In the multivariate logistic regression model, both CAR and RAR were positively correlated with the incidence of nutritional risk. For every one-unit increase in CAR, there were 50.2% higher odds of having a moderate to severe risk of PEW (as reflected by MIS) (OR = 1.502; 95% CI: 1.22–1.85; *p* < 0.001; Table 2). For every one-unit increase in RAR, the risk of occurrence increased by 26.3% (OR = 1.263; 95% CI: 1.04–1.54; *p* = 0.019; Table 2).

**TABLE 2:** Univariate and multivariate logistic regression analyses of factors associated with high malnutrition-inflammation score (MIS) in maintenance hemodialysis patients.

	Univariate analysis		Multivariate analysis	
Covariates	OR (95% CI)	*p*-value	Adjusted OR (95% CI)	*p-*value
Age	1.042(1.020–1.064)	< 0.001		
Sex				
Famale	1	0.675		
Male	1.120(0.659–1.906)			
Height	0.980(0.947–1.014)	0.243		
Kt/V	1.381(0.759–2.512)	0.291		
Dialysis age	0.910(0.847–0.977)	0.01		
Dry weight	1.000(0.979–1.020)	0.951		
BMI	1.036(0.969–1.106)	0.301		
DM				
No	1	0.045		
Yes	1.773(1.013–3.106)			
CRP	1.152(1.092–1.216)	< 0.001		
ALB	0.025(0.007–0.089)	< 0.001		
CAR	1.670(1.370–2.035)	< 0.001	1.502(1.218–1.852)	< 0.001
RAR	1.582(1.334–1.877)	< 0.001	1.263(1.040–1.535)	0.019
PAR	1.011(0.996–1.026)	0.165		

Variables with a *p* < 0.05 in the univariate analysis were included in the multivariate model. The variance inflation factor (VIF) was calculated to adjust for multicollinearity. OR, odds ratio; CI, confidence interval; Kt/V, dialysis adequacy; BMI, body mass index; DM, diabetes mellitus; CRP, C-reactive protein; ALB, albumin; CAR, C-reactive protein to albumin ratio; RAR, red cell distribution width to albumin ratio; PAR, platelet to albumin ratio. *p* < 0.05 was considered statistically significant.

### 3.3 ROC curve analysis

**FIGURE 2:**
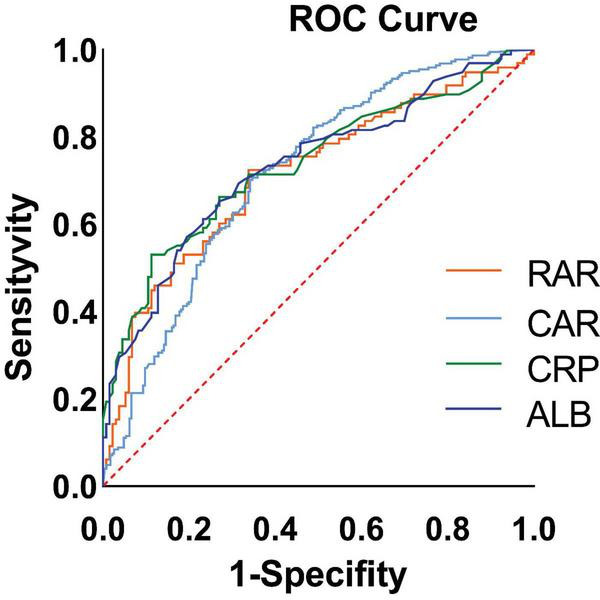
Receiver operating characteristic (ROC) curves of biomarkers for predicting high malnutrition-inflammation score (MIS) in maintenance hemodialysis (MHD) patients. The ROC curves compare the performance of the C-reactive protein to albumin ratio (CAR), the red blood cell distribution width to albumin ratio (RAR), C-reactive protein (CRP) alone, and albumin (ALB) alone. The diagonal line represents the reference line of no discriminative ability (AUC = 0.5). The area under the curve (AUC) for each biomarker is presented in the inset table alongside its optimal cutoff value, sensitivity, and specificity. CAR demonstrated the highest AUC (73.85%, 95% CI: 67.17–80.53%) for identifying patients with MIS ≥ 7.

Next, the ROC curves for CAR, RAR, C-reactive protein, and serum albumin were plotted (Figure 2). Their predictive performance is shown in Table 3. The CAR yielded the best predictive performance, with an area under the curve (AUC) of 73.85% (95% CI: 67.17–80.53%), and an optimal cut-off value of 2.158, associated with a sensitivity of 53.06% and a specificity of 88.72%.

**TABLE 3:** Predictive performance of biomarkers for high malnutrition-inflammation score (MIS).

Biomarker	Sensitivity (%)	Specificity (%)	AUC (95%CI)	Youden index	Cut-off	*p*-value
CAR	53.06	88.72	73.85(67.17–80.53)	0.418	2.158	< 0.0001
RAR	72.45	66.17	71.56(64.71–78.41)	0.386	12.63	< 0.0001
CRP	53.06	88.72	73.71(67.01–50.40)	0.418	8	< 0.0001
ALB	65.31	72.93	73.48(66.85–80.11)	0.382	3.845	< 0.0001

The area under the curve (AUC), optimal cut-off value, sensitivity, specificity, and Youden index for each biomarker are shown. CAR, C-reactive protein to albumin ratio; RAR, red cell distribution width to albumin ratio; CRP, C-reactive protein; ALB, albumin; AUC, area under the curve; CI, confidence interval.

### 3.4 Non-linear relationship between CAR and MIS score and its threshold

**FIGURE 3:**
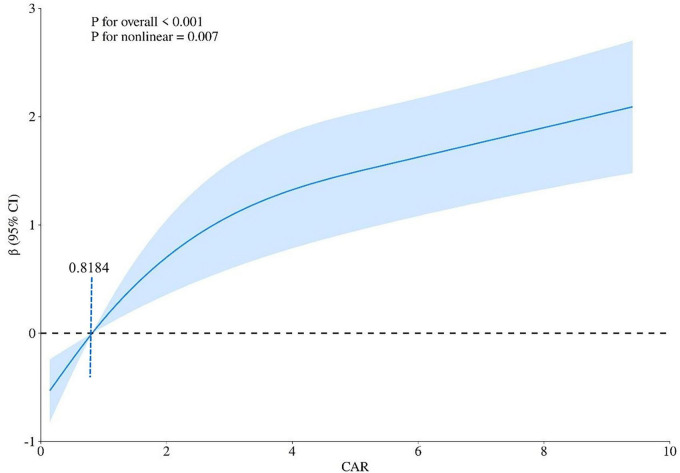
Non-linear association between the C-reactive protein to albumin ratio (CAR) and the malnutrition-inflammation score (MIS) evaluated by restricted cubic spline (RCS) regression. The solid curve represents the estimated odds ratio (OR) for high MIS associated with CAR levels, with the reference point set at the median CAR value. The shaded area represents the 95% confidence interval. The non-linear relationship was statistically significant (*p* for non-linearity = 0.007). Two distinct phases were identified: a negative association at lower CAR values (< 0.8184, the inflection point) and a positive association at higher CAR values (≥ 0.8184).

An RCS curve was plotted to clarify the non-linear relationship between MIS and CAR (*p* = 0.007). The relationship demonstrated an inflection point at a CAR value of 0.8184, with a negative correlation observed below this threshold and a positive correlation above it (Figure 3).

## 4 Discussion

This cross-sectional study was conducted to investigate PEW in MHD patients at our medical center, with the primary objective of elucidating the intricate associations among the CAR, nutritional status, and inflammation. The MIS was employed to comprehensively assess the nutritional and inflammatory risks of the patients. Notably, a significant independent correlation was identified between CAR levels and the presence of nutritional and inflammatory risks in MHD patients. Specifically, each unit increase in CAR was associated with 50.2% higher odds (OR = 1.502) of nutritional risk (as defined by MIS). Importantly, even after adjustment for potential multicollinearity, this correlation remained statistically significant and robust, demonstrating the reliability of our findings. To further evaluate the predictive capacity of CAR for nutritional risk, an ROC curve was constructed. Among the biomarkers evaluated, CAR demonstrated the highest predictive value for MIS, with an AUC of 73.85% (95% CI: 67.17–80.53%), indicating moderate discriminative ability. ROC analysis revealed that CAR possesses high specificity (88.72%) but modest sensitivity (53.06%) at its optimal cutoff. This performance profile underscores its potential role and limitations within the context of a cross-sectional association. The high specificity indicates that an elevated CAR (≥ 2.158) is strongly associated with a high MIS score, effectively helping to identify the risk of PEW with a low false-positive rate. This associative strength suggests CAR could be a useful adjunctive measure for confirming the presence of PEW in clinical practice. Conversely, its suboptimal sensitivity indicates that a low CAR value cannot reliably rule out a high MIS, as nearly half of the patients with high scores would not be identified by this marker alone, given that the MIS incorporates multifaceted components beyond systemic inflammation and albumin status. Therefore, while a significant cross-sectional association exists, our study design cannot establish causality, and CAR should not be interpreted as a causative factor or a standalone screening tool. Instead, its utility may lie in being part of a broader multimodal assessment strategy to estimate the likelihood of protein-energy wasting. In addition, restricted cubic spline analysis revealed a non-linear relationship between the CAR and nutritional risk. When the CAR values were stratified using a threshold of 0.8184, a distinct bifurcation in the correlation emerged. Specifically, when CAR levels were below 0.8184, an increase in CAR was associated with a decreasing probability of nutritional risk. Conversely, once CAR reached or exceeded 0.8184, a positive correlation was observed, where increasing CAR values corresponded to a higher likelihood of nutritional risk. This observation highlights a non-linear relationship between CAR and nutritional status in MHD patients. Collectively, these findings highlight the potential of CAR as a valuable biomarker for predicting nutritional risk in MHD patients, providing valuable information for the management of nutritional status in this patient population.

Hemodialysis is one of the most significant renal replacement therapies for patients with stage 5 chronic kidney disease (CKD). It is now understood that energy consumption is increased during the hemodialysis process. The International Society of Renal Nutrition and Metabolism has introduced the term “protein-energy wasting-PEW” for CKD patients (13). Numerous studies on CKD have indicated that PEW in MHD patients is associated with cachexia, adverse cardiovascular events, and all-cause mortality (29, 30).

Besides, MHD patients often experience a chronic systemic inflammatory state, primarily mediated by multiple mechanisms, including the accumulation of uremic toxins, biocompatibility issues inherent to dialysis procedures (such as complement activation cascades and endotoxin translocation), and heightened susceptibility to infectious complications (3–5), combined with cardiovascular and cerebrovascular diseases and other underlying conditions (31). The inflammatory response activates the NF-κB pathway, inducing the release of pro-inflammatory cytokines such as tumor necrosis factor-α (TNF-α) and interleukin-6 (IL-6). On one hand, these cytokines directly inhibit the transcription of the liver albumin gene—for example, by downregulating the nuclear activity of CCAAT/enhancer-binding protein-β (C/EBP-β), blocking the synthesis of albumin mRNA (32, 33). On the other hand, it accelerates muscle protein breakdown by activating the ubiquitin-proteasome system (34, 35). In addition, inflammation-induced vascular endothelial injury increases vascular permeability, causing albumin to leak from the blood vessels into the tissue space, further reducing the circulating albumin level (36). Moreover, the establishment of dialysis-related accesses poses a risk of catheter-related infections. In an inflammatory state, the body undergoes high catabolism, with accelerated breakdown of muscle proteins, which further exacerbates energy consumption and increases the risk of all-cause adverse events in patients (37–39).

Timely identification of nutritional-inflammatory risks in MHD patients is a crucial part of chronic disease management. However, the laboratory examination process for hemodialysis patients needs to balance practicality and economic benefits. Therefore, using a combination of various biomarkers reflecting nutritional and inflammatory processes is a valuable approach. Previous studies have confirmed the roles of various inflammatory biomarkers and their ratios in clinical applications. These include single inflammatory indicators such as CRP, tumor necrosis factor-β, procalcitonin, and IL-6 (39–41), and nutritional indicators such as BMI, albumin, and pre-albumin (42–44). In recent years, it has been found that albumin and pre-albumin are not only widely used as nutritional indicators but also important inflammatory indicators, and they have been proven to have significant value in predicting mortality in CKD patients. Composite inflammatory indicators such as the neutrophil-lymphocyte ratio (NLR), platelet-lymphocyte ratio (PLR), and systemic immune-inflammation index (SII) (38), and inflammation-nutrition indicators including the RAR, fibrinogen to albumin ratio (FAR), and CAR (45, 46).

Prior research has identified an independent association between the CAR and the MIS in patients undergoing dialysis (28). Our findings are consistent with those reported by Tur and Güçlü, who also identified an independent association between CAR and MIS in a cohort of hemodialysis patients. However, our study extends their work by employing a larger sample size (*n* = 231 vs. *n* = 120) and utilizing restricted cubic spline analysis to reveal a non-linear relationship between CAR and MIS, with a distinct threshold effect at CAR = 0.8184. This threshold may represent a metabolic shift point where inflammatory processes may start to substantially affect nutritional status, a nuance not previously captured in linear analyses.

Moreover, while Tur and Güçlü focused primarily on the linear correlation, our ROC analysis provided a clinically actionable cut-off value (CAR = 2.158) with high specificity, suggesting that CAR could serve as a useful screening tool in settings where comprehensive nutritional assessment is not immediately feasible. Nevertheless, the modest sensitivity of CAR underscores the necessity of integrating it with other biomarkers or clinical scores for a more robust evaluation of PEW.

Existing nutritional scoring systems for dialysis patients, including MIS, Subjective Global Assessment (SGA), Nutritional Risk Screening 2002 (NRS 2002), Kalantar score, and Prognostic Nutritional Index (PNI) (47–51), integrate multiple indicators. These calculations are complex and difficult to derive in a timely manner. However, according to the Chinese Standard Operating Procedures for Blood Purification, it is essential to routinely monitor the biochemical indicators and CRP levels in dialysis patients to efficiently and conveniently calculate the malnutrition risk index, known as the CAR. The CAR demonstrates definitive predictive value in assessing nutritional and inflammatory risks. In the context of chronic disease management, the early identification of patients at nutritional risk is crucial, and the clinical utility of CAR measurement in guiding therapeutic decisions is significant. Given its affordability and ease of access, CAR may serve as a practical biomarker associated with nutritional risk, potentially complementing existing multi-factor scoring systems in clinical practice. Consequently, it could provide a more convenient approach for evaluating the nutritional and inflammatory risks in patients undergoing MHD.

This study has several limitations that should be acknowledged. Firstly, as a single-center study, the sample was exclusively sourced from the Affiliated Hospital of Southwest Medical University, lacking external validation from independent patient cohorts. This limitation may compromise the generalizability of the findings, as the results may not be applicable to MHD patients from diverse geographical regions and healthcare settings. Secondly, the cross-sectional study design employed in this research precludes the establishment of a causal relationship between the CAR and the MIS score. Since the measurements of CAR levels and MIS scores were conducted simultaneously, it is challenging to determine whether changes in CAR lead to alterations in the nutritional and inflammatory status (as reflected by the MIS score), or vice versa. Moreover, it is imperative to acknowledge the potential influence of unmeasured confounding variables. These include heterogeneity in underlying etiology, the impact of comorbidities [e.g., heart failure, chronic obstructive pulmonary disease (COPD)] and medications (e.g., immunosuppressants, corticosteroids) on inflammatory modulation, and a lack of comprehensive data on dietary intake—a core determinant of nutritional status. Furthermore, sources of inflammation unrelated to the malnutrition-inflammation axis (e.g., occult infection, gut dysbiosis) may independently elevate CAR. Indeed, these unaccounted-for factors may influence the interpreted independence of the CAR-MIS relationship.

## 5 Conclusion

In chronic disease management, early identification of patients at nutritional risk is of utmost importance. The clinical utility of CAR measurement in guiding treatment decisions is notable. Considering its low cost and easy accessibility, CAR may prove to be a practical complementary tool to complex multi-factor scoring systems in clinical practice. This makes it a more convenient option for rapidly evaluating the nutritional risk of MHD patients.

## Data Availability

The raw data supporting the conclusions of this article will be made available by the authors, without undue reservation.

## Footnotes

1
www.zstats.net

## References

[1] Sá Martins V Aguiar L Dias C Lourenço P Pinheiro T Velez B Predictors of nutritional and inflammation risk in hemodialysis patients. *Clin Nutr.* (2020) 39:1878–84. 10.1016/j.clnu.2019.07.029 31427179

[2] Bramania P Ruggajo P Bramania R Mahmoud M Furia F. Prevalence of malnutrition inflammation complex syndrome among patients on maintenance haemodialysis at Muhimbili National Hospital in Tanzania: a cross-sectional study. *BMC Nephrol.* (2020) 21:521. 10.1186/s12882-020-02171-3 33256618 PMC7708158

[3] Castillo-Rodríguez E Pizarro-Sánchez S Sanz A Ramos A Sanchez-Niño M Martin-Cleary C Inflammatory cytokines as uremic toxins: “Ni Son Todos Los Que Estan, Ni Estan Todos Los Que Son”. *Toxins.* (2017) 9:114. 10.3390/toxins9040114 28333114 PMC5408188

[4] Ekdahl K Soveri I Hilborn J Fellström B Nilsson B. Cardiovascular disease in haemodialysis: role of the intravascular innate immune system. *Nat Rev Nephrol.* (2017) 13:285–96. 10.1038/nrneph.2017.17 28239169

[5] Craddock P Fehr J Brigham K Kronenberg R Jacob H. Complement and leukocyte-mediated pulmonary dysfunction in hemodialysis. *N Engl J Med.* (1977) 296:769–74. 10.1056/NEJM197704072961401 840277

[6] Kalantar-Zadeh KI Block G Avram M Kopple J. Malnutrition-inflammation complex syndrome in dialysis patients: causes and consequences. *Am J Kidney Dis.* (2003) 42:864–81. 10.1053/S0272-6386(03)01005-9 14582032

[7] Hynote, McCamish M Depner T Davis P. Amino acid losses during hemodialysis: effects of high-solute flux and parenteral nutrition in acute renal failure. *J Parenteral Enteral Nutr.* (1995) 19:15–21. 10.1177/014860719501900115 7658594

[8] March D Graham-Brown M Stover C Bishop N Burton J. Intestinal barrier disturbances in haemodialysis patients: mechanisms, consequences, and therapeutic options. *BioMed Res Int.* (2017) 2017:1–11. 10.1155/2017/5765417 28194419 PMC5282437

[9] Vaziri N Zhao Y Pahl M. Altered intestinal microbial flora and impaired epithelial barrier structure and function in CKD: the nature, mechanisms, consequences and potential treatment. *Nephrol Dialysis Transpl.* (2016) 31:737–46. 10.1093/ndt/gfv095 25883197

[10] Kaysen G. Progressive inflammation and wasting in patients with ESRD. *Clin J Am Soc Nephrol.* (2014) 9:225–6. 10.2215/cjn.12541213 24458072 PMC3913250

[11] Tabata F Wada Y Kawakami S Miyaji K. Serum albumin redox states: more than oxidative stress biomarker. *Antioxidants.* (2021) 10:503. 10.3390/antiox10040503 33804859 PMC8063786

[12] Morgan M Liu Z. Crosstalk of reactive oxygen species and NF-κB signaling. *Cell Res.* (2010) 21:103–15. 10.1038/cr.2010.178 21187859 PMC3193400

[13] Fouque D Kalantar-Zadeh K Kopple J Cano N Chauveau P Cuppari L A proposed nomenclature and diagnostic criteria for protein–energy wasting in acute and chronic kidney disease. *Kidney Int.* (2008) 73:391–8. 10.1038/sj.ki.5002585 18094682

[14] Carrero J Thomas F Nagy K Arogundade F Avesani C Chan M Global prevalence of protein-energy wasting in kidney disease: a meta-analysis of contemporary observational studies from the international society of renal nutrition and metabolism. *J Renal Nutr.* (2018) 28:380–92. 10.1053/j.jrn.2018.08.006 30348259

[15] Kovesdy C. Malnutrition in dialysis patients—the need for intervention despite uncertain benefits. *Semin Dialysis.* (2015) 29:28–34. 10.1111/sdi.12410 26190025

[16] Graterol Torres F Molina M Soler-Majoral J Romero-González G Rodríguez Chitiva N Troya-Saborido M Evolving concepts on inflammatory biomarkers and malnutrition in chronic kidney disease. *Nutrients.* (2022) 14:4297. 10.3390/nu14204297 36296981 PMC9611115

[17] Balali A Nehls M Tabibi H As’habi A Arab A. Dietary acid load and markers of malnutrition, inflammation, and oxidative stress in hemodialysis patients. *Front Nutr.* (2024) 11:1369206. 10.3389/fnut.2024.1369206 38585612 PMC10998450

[18] Yamada S Yamamoto S Fukuma S Nakano T Tsuruya K Inaba M. Geriatric nutritional risk index (GNRI) and creatinine index equally predict the risk of mortality in hemodialysis patients: J-DOPPS. *Sci Rep.* (2020) 10:5756. 10.1038/s41598-020-62720-6 32238848 PMC7113241

[19] Arias-Guillén M Collado S Coll E Carreras J Betancourt L Romano B Prevalence of protein-energy wasting in dialysis patients using a practical online tool to compare with other nutritional scores: results of the nutrendial study. *Nutrients.* (2022) 14:3375. 10.3390/nu14163375 36014879 PMC9413877

[20] Andreas Eckart MD Alexander Kutz MD Annic Baumgartner MD Seline Zurfluh MD Neeser O Andreas Huber MD Relationship of nutritional status, inflammation, and serum albumin levels during acute illness: a prospective study. *Am J Med.* (2020) 133: 713–22.e7. 10.1016/j.amjmed.2019.10.031 31751531

[21] Soeters PW Shenkin A. Hypoalbuminemia: pathogenesis and clinical significance. *JPEN J Parenter Enteral Nutr.* (2019) 43:181–93. 10.1002/jpen.1451 30288759 PMC7379941

[22] Sheinenzon A Shehadeh M Michelis R Shaoul E Ronen O. Serum albumin levels and inflammation. *Int J Biol Macromol.* (2021) 184:857–62. 10.1016/j.ijbiomac.2021.06.140 34181998

[23] Hutson A Zhou W Zhang GL. C-reactive protein to albumin ratio predicts the outcome in renal cell carcinoma: a meta-analysis. *PLoS One.* (2019) 14:e0224266. 10.1371/journal.pone.0224266 31644587 PMC6808556

[24] Liu Y Gao Y Liang B Liang Z. The prognostic value of C-reactive protein to albumin ratio in patients with sepsis: a systematic review and meta-analysis. *Aging Male.* (2023) 26:2261540. 10.1080/13685538.2023.2261540 37752726

[25] Chen S Guan S Yan Z Ouyang F Li S Liu L Prognostic value of red blood cell distribution width-to-albumin ratio in ICU patients with coronary heart disease and diabetes mellitus. *Front Endocrinol.* (2024) 15:1359345. 10.3389/fendo.2024.1359345 39387054 PMC11461254

[26] Tan J Song G Wang S Dong L Liu X Jiang Z Platelet-to-albumin ratio: a novel IgA nephropathy prognosis predictor. *Front Immunol.* (2022) 13:842362. 10.3389/fimmu.2022.842362 35664006 PMC9162245

[27] Borges M Vogt B Martin L Caramori J. Malnutrition inflammation score cut-off predicting mortality in maintenance hemodialysis patients. *Clin Nutr ESPEN.* (2017) 17:63–7. 10.1016/j.clnesp.2016.10.006 28361749

[28] Tur K Güçlü A. Independent association between malnutrition inflammation score and C reactive protein/albumin ratio in hemodialysis patients. *J Inflammation Res.* (2024) 17:9325–33. 10.2147/jir.S477307 39600674 PMC11590669

[29] Kim J Kalantar-Zadeh K Kopple J. Frailty and protein-energy wasting in elderly patients with end stage kidney disease. *J Am Soc Nephrol.* (2013) 24:337–51. 10.1681/asn.2012010047 23264684

[30] Carrero J Nakashima A Qureshi A Lindholm B Heimbürger O Bárány P Protein-energy wasting modifies the association of ghrelin with inflammation, leptin, and mortality in hemodialysis patients. *Kidney Int.* (2011) 79:749–56. 10.1038/ki.2010.487 21178976

[31] Jofré R Rodriguez-Benitez P López-Gómez JM Pérez-Garcia R. Inflammatory syndrome in patients on hemodialysis. *J Am Soc Nephrol.* (2006) 17:S274–80. 10.1681/ASN.2006080926 17130274

[32] Chojkier M. Inhibition of albumin synthesis in chronic diseases molecular mechanisms. *J Clin Gastroenterol.* (2005) 39:S143–6. 10.1097/01.mcg.0000155514.17715.39 15758650

[33] Ehlting C Wolf S Bode J. Acute-phase protein synthesis: a key feature of innate immune functions of the liver. *Biol Chem.* (2021) 402:1129–45. 10.1515/hsz-2021-0209 34323429

[34] Pang X Zhang P Chen X Liu W. Ubiquitin-proteasome pathway in skeletal muscle atrophy. *Front Physiol.* (2023) 14:1289537. 10.3389/fphys.2023.1289537 38046952 PMC10690626

[35] Haberecht-Müller S Krüger E Fielitz J. Out of control: the role of the ubiquitin proteasome system in skeletal muscle during inflammation. *Biomolecules.* (2021) 11:1327. 10.3390/biom11091327 34572540 PMC8468834

[36] Malyszko J. Mechanism of endothelial dysfunction in chronic kidney disease. *Clinica Chimica Acta.* (2010) 411:1412–20. 10.1016/j.cca.2010.06.019 20598675

[37] Alessandro Amore R. Immunological basis of inflammation in dialysis. *Nephrol Dial Transplant.* (2002) 17:16–24. 10.1093/ndt/17.suppl_8.16 12147772

[38] Chen M Wu J Ma H Luo L Liu L Zhang J Association between complex indices of blood cell types and lipid levels with all-cause, cardiovascular mortality in hemodialysis patients: a multicenter retrospective study. *Ther Apher Dial.* (2025) 29:345–56. 10.1111/1744-9987.70010 40129084

[39] Sasaki K Shoji T Kabata D Shintani A Okute Y Tsuchikura S Oxidative stress and inflammation as predictors of mortality and cardiovascular events in hemodialysis patients: the DREAM cohort. *J Atherosc Thrombosis.* (2021) 28:249–60. 10.5551/jat.56069 32741893 PMC8049144

[40] Beberashvili I Sinuani I Azar A Kadoshi H Shapiro G Feldman L Decreased IGF-1 levels potentiate association of inflammation with all-cause and cardiovascular mortality in prevalent hemodialysis patients. *Growth Hormone IGF Res.* (2013) 23:209–14. 10.1016/j.ghir.2013.07.005 23958273

[41] Brankovic M Kim I Kim S Ye B Kim M Kim S Procalcitonin decrease predicts survival and recovery from dialysis at 28 days in patients with sepsis-induced acute kidney injury receiving continuous renal replacement therapy. *PLoS One.* (2022) 17:e0279561. 10.1371/journal.pone.0279561 36574383 PMC9794048

[42] Suzuki Y Harada M Matsuzawa R Hoshi K Koh Y Aoyama N Trajectory of serum albumin prior to death in patients receiving hemodialysis. *J Renal Nutr.* (2023) 33:368–75. 10.1053/j.jrn.2022.07.007 36007716

[43] Okuno S. Significance of adipose tissue maintenance in patients undergoing hemodialysis. *Nutrients.* (2021) 13:1895. 10.3390/nu13061895 34072922 PMC8226793

[44] Pardo E Jabaudon M Godet T Pereira B Morand D Futier E Dynamic assessment of prealbumin for nutrition support effectiveness in critically ill patients. *Clin Nutr.* (2024) 43:1343–52. 10.1016/j.clnu.2024.04.015 38677045

[45] Hao M Jiang S Tang J Li X Wang S Li Y Ratio of red blood cell distribution width to albumin level and risk of mortality. *JAMA Netw Open.* (2024) 7:e2413213. 10.1001/jamanetworkopen.2024.13213 38805227 PMC11134218

[46] Zou Y Zhu Z Zhou J Wu X Li H Ning X Fibrinogen/Albumin ratio: a more powerful prognostic index for patients with end-stage renal disease. *Eur J Clin Invest.* (2020) 50:e13266. 10.1111/eci.13266 32379901

[47] Hanna Ramy M Ghobry L Wassef O Rhee Connie M Kalantar-Zadeh K. A practical approach to nutrition, protein-energy wasting, sarcopenia, and cachexia in patients with chronic kidney disease. *Blood Purification.* (2020) 49:202–11. 10.1159/000504240 31851983

[48] Brandão da Cunha Bandeira S Cansanção K Pereira de Paula T Peres WAF. Evaluation of the prognostic significance of the malnutrition inflammation score in hemodialysis patients. *Clin Nutr ESPEN.* (2020) 35:109–15. 10.1016/j.clnesp.2019.10.019 31987102

[49] Sá Martins V Adragão T Aguiar L Pinto I Dias C Figueiredo R Prognostic value of the malnutrition-inflammation score in hospitalization and mortality on long-term hemodialysis. *J Renal Nutr.* (2022) 32:569–77. 10.1053/j.jrn.2021.11.002 34922814

[50] Liu Y Wang L Li S Xu S Zhou D Zhong X Associations between blood trace element levels and nutritional status in maintenance hemodialysis. *J Renal Nutr.* (2021) 31:661–8. 10.1053/j.jrn.2020.12.007 33941438

[51] Miyasato Y Hanna R Morinaga J Mukoyama M Kalantar-Zadeh K. Prognostic nutritional index as a predictor of mortality in 101,616 patients undergoing hemodialysis. *Nutrients.* (2023) 15:311. 10.3390/nu15020311 36678182 PMC9865495

